# Ferulic acid promoting apoptosis in human osteosarcoma cell lines

**DOI:** 10.12669/pjms.331.12066

**Published:** 2017

**Authors:** Xu-dong Zhang, Qiang Wu, Shu-hua Yang

**Affiliations:** 1 Dr. Xu-dong Zhang, Department of Orthopedics, Union Hospital, Tongji Medical College, Huazhong University of Science and Technology, Wuhan, China; 2 Prof. Qiang Wu, Department of Orthopedics, Union Hospital, Tongji Medical College, Huazhong University of Science and Technology, Wuhan, China; 3 Prof. Shu-hua Yang, Department of Orthopedics, Union Hospital, Tongji Medical College, Huazhong University of Science and Technology, Wuhan, China

**Keywords:** Apoptosis, Bcl-2, Bax, Caspase-3, Ferulic acid, Procaspase-3, Osteosarcoma

## Abstract

**Objective:**

To explore the promoting apoptosis and antitumor activities of ferulic acid (FA) in human osteosarcoma and its potential mechanism.

**Methods:**

The SaOS-2 and MG63 osteosarcoma cell lines were opted to experiment and these cells were, respectively, cultured with various concentrations of FA (0 μM, 10 μM, 20 μM, 40 μM) for 72 hours at 37°C. The viabilities of the FA treated cells were monitored by MTT. Apoptosis cells were evaluated using annexin V/PI by flow cytometry. Apoptosis proteins caspase-3, procaspase-3, Bcl-2 and Bax were detected by western blot. Expressions of apoptotic genes Bcl-2 and Bax were quantified by qPCR.

**Results:**

The cell viabilities were critically declined in the concentration-dependent manner in FA groups (*P* < 0.01). The apoptosis cells were increased proportionately with the concentration of FA (*P* < 0.05). The procaspase-3 protein contents, and Bcl-2 mRNA and protein contents were significantly decreased while caspase-3 protein contents, and Bax mRNA and protein contents were concomitantly increased in the concentration-dependent manner in FA groups (*P* < 0.05). The response to FA by the SaOS-2 osteosarcoma cell was similar with the MG63 osteosarcoma cell (*P* > 0.05).

**Conclusion:**

Ferulic acid could significantly descend osteosarcoma cell viability through the promoting apoptosis pathway in which FA activates both caspase-3 and Bax and inactivates Bcl-2.

## INTRODUCTION

Osteosarcoma, an intra-osseous malignant neoplasm, has a bimodal age distribution of incidence which is comprised of an adolescent peak encountered in about age 15 and an elderly peak encountered in about age 75.^1^ The prognoses of patients diagnosed osteosarcoma are really difficult to satisfy, albeit in a somewhat improvement over the past several decades indebted to the introduction of surgical removal and neoadjuvant chemotherapy.^2^ The development of effective and efficient therapeutic strategies is urgently required.

FA (4-hydroxy-3-methoxycinnamic acid) is extracted from some natural plants, such as *Asafoetida giantfennel, Angelica sinensis, Cimicifuga racemosa, Glycyrrhiza uralensis*, and *Ligusticum chuanxiong*, which have been employed as the traditional Chinese herbs to treat multiple diseases for several thousand years.^3^-^5^ Recently, numerous literatures have demonstrated the antitumor activities and protective effects of FA on thyroid cancer, prostate cancer, skin carcinogenesis, lung cancer, etc.^6^-^9^ Whereas the antitumor activities and its mechanism of FA in osteosarcoma remain still under discussion.

Apoptosis, ultimately resulting in an active and programmed demise of the cell, is originally induced by all sorts of physiological and pathological stimuli, including a broad variety of naturally occurring molecules, especially extractions from plants. FA has been reported to induce apoptosis in TT thyroid cancer cells and LNCaP prostate cancer cells.^6^,^7^ However, whether FA could promote apoptosis in osteosarcoma is still mysterious. In this study we investigate the influences of FA on apoptosis in osteosarcoma cell lines.

## METHODS

FA, DMEM medium, fetal calf serum (FCS), penicillin, streptomycin, EDTA solution and other related reagents were obtained from Sigma Chemicals Co. FA were dissolved and diluted with PBS to the indicated concentration. Annexin V/PI apoptosis kit, caspase-3 antibody, Bax antibody, Bcl-2 antibody, Gapdh antibody, RT-PCR kit, and other related reagents were purchased from Oncogene.

The SaOS-2 and MG63 human osteosarcoma cell lines were purchased from China Center for Type Culture Collection. The cells were cultured in DMEM medium plus 10% FCS, 100 IU/ml penicillin and 50 μg/ml streptomycin at 37°C in the environments containing 5% CO_2_ for 24 hours prior to experimentation. Then the cells were further cultured with various concentrations of FA (0 μM, 10 μM, 20 μM, 40 μM) for 72 hours at 37°C.

### Cell viabilities monitored by MTT

The cell suspension containing 1×10^6^ cells/ml were cultured in a 96 wells culture plate (100 μl cells suspension per well) overnight and further incubated with 10 μl MTT (10 mg/ml) for 4 hours at 37°C. The medium were centrifuged for 5 minutes at 1000 rpm in order to discarding supernatant and the crystals were fully dissolved with 200 μl DMSO after 20 minutes oscillation. The optical density (OD) of per well was measured with a spectrophotometer (DYNEX Technologies, Denkendorf, Germany) at a wavelength of 570 nm.

### Apoptosis cell evaluated by flow cytometry

Approximately 2 x 10^6^ cells collected and washed twice with ice-cold PBS solution were resuspended in 500 μl of binding buffer. The cell suspension was incubated with 10 μl Annexin V-FITC (20 μg/ml) and 5 μl PI (50 μg/ml) for 30 minutes at 20°C in the darkness. The quantities of annexin V-positive cells of samples were immediately detected and calculated by flow cytometry (FACScan, Becton Dickinson, Mountain View, CA, USA).

### Apoptosis protein detected by western blot

The cultured cells were harvested and lysed with 200 μl ice-cold PBS plus 1% Triton X-100, 0.1% SDS, 0.5% sodium deoxycholate, 10 mg/ml aprotinin, 10 mg/ml leupeptin, and 1 mM phenylmethylsulfonylfluoride. After centrifugation at 10,000 rpm at 4°C for 10 minutes, the supernatant protein was quantified by the Lowry method. Then 20 μg protein were mixed with SDS-PAGE sample buffer and mercaptoethanol to incubation at 100°C for 5 minutes, separated by 15% SDS-PAGE and transferred to PVDF membrane. The membranes blocked in 5% fat-free milk were incubated with 1:2,000 rabbit polyclonal antihuman caspase-3 antibody, Bax antibody, Bcl-2 antibody, and Gapdh antibody at 20°C for two hours and then with 1:3,000 HRP conjugated secondary antibody at 20°C for one hours. After membrane was thrice washed in TBST for interval of 10 minutes, bands of procaspase-3, caspase-3, Bax, Bcl-2, or Gapdh protein were visualized with ECL chromogenic substrate. The relative levels of these proteins to control Gapdh were probed with image J software.

### Apoptotic genes expression quantified by qPCR

Total RNA were isolated from the cultured cells using Trizol reagent, then quantified to 1μg/μl by spectrophotometer. First strand cDNA was prepared using reverse transcription reaction system: 1 μl total RNA, 1 μl reverse transcriptase, 1 μl oligo (DT), 4 μl 5×RTase buffer, 1 μl Rnasin, 3 μl MgCl_2_ (25 mM), 2 μl dNTPs (10 mM), and 7 μl DEPC treated water, then incubated at 37°C for 60 minutes and inactivated at 93°C for 10 minutes. Bax or Bcl-2 mRNA levels of each cell lines were quantified by quantitative real-time PCR (qPCR) utilizing RT-PCR kits, in line with the manufacturer’s instruction. The primers were designed per PRIMER 5.0 software and synthesized by Sangon Biotech. For Bax, the PCR primers were forward primer 5’-TTTGCTTCAGGGTTTCATCC-3’ and reverse primer 5’-AGACCTGCCGTTGAAGTTGAC-3’. For Bcl-2, the PCR primers were forward primer 5’-GGATGCCTTTGTGGAACTGT-3’ and reverse primer 5’-TACTTTGTTTCGACGTCCGA-3’. For Gapdh, the PCR primers were forward primer 5’-TTCACCACCATGGAGAAGGC-3’ and reverse primer 5’-GGCATGGACTGTGGTCATGA-3’. qPCR analysis was performed with cDNA reaction as a template, utilizing the StepOnePlus Real-Time PCR System (Applied Biosystems, Foster City, CA, USA). The ΔΔCT method using Gapdh as a reference was utilized to compute the relative expression of Bcl-2 or Bax. The procedure was entirely repeated thrice using independent samples.

### Statistical Analysis

SPSS 15.0 software package was utilized for statistical analysis. Statistical significance was determined using one-way ANOVA. The *P* value less than 0.05 was considered to be statistically significant.

## RESULTS

**Fig. 1:**
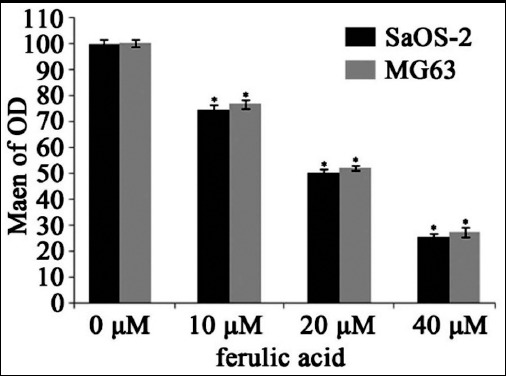
The cell viabilities of osteosarcoma cell lines after exposure to FA.

The OD values of the various concentrations of FA group were gradually diminished. The viability and metabolism of cells were significantly descended in the concentration-dependent manner. Cells of the control group (0 μM FA) still maintain higher viability and metabolism. There were significant differences in four groups of each cell line (*P* < 0.01). (Fig.1)

**Fig. 2:**
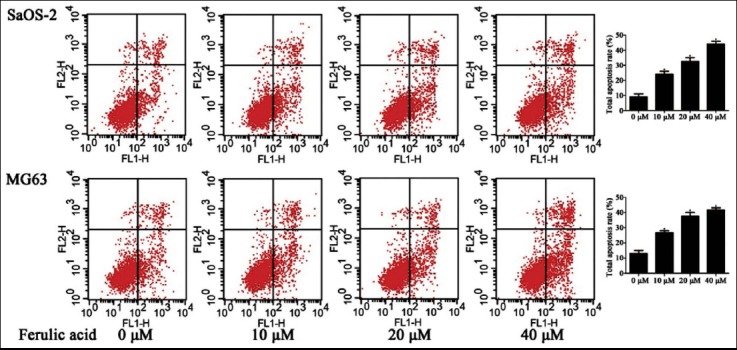
The apoptotic cells of osteosarcoma cell lines treated to FA.

The apoptotic cells detected by flow cytometry had demonstrated that there were significantly increased in the population of apoptotic cells of the FA group compared to the control group (*P* < 0.05). The apoptotic cells treated to various concentrations of FA had gradually increased in the concentration-dependent manner (*P* < 0.05). (Fig.2)

**Fig. 3:**
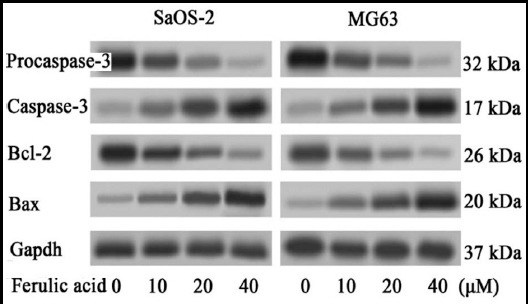
The apoptotic protein procaspase-3, caspase-3, Bcl-2 and Bax in osteosarcoma cell lines cultured with FA.

At 72 hours post-exposure, apoptotic protein procaspase-3 holoenzyme was activated and cleaved the p17 cleavage product, which were both recognized by a caspase-3 antibody. The quantities of the 32 kDa procaspase-3 protein were significantly decreased and the 17 kDa caspase-3 protein contents were simultaneously increased in FA group compared to the control group (*P* < 0.05). In some similar manner, Bax protein contents were significantly increased and Bcl-2 protein contents were concomitantly decreased in FA group compared to the control group (*P* < 0.05). All changes were happened in the concentration-dependent manner. (Fig.3)

**Fig. 4:**
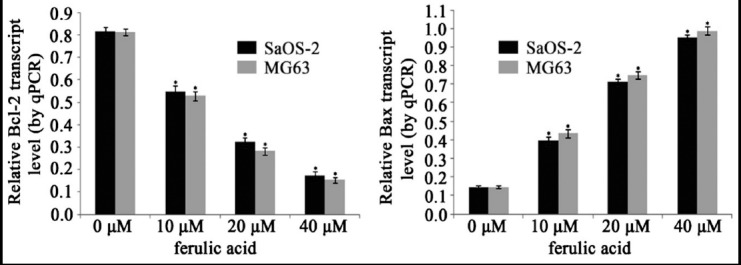
The expression of Bcl-2 and Bax in osteosarcoma cell lines exposed to FA.

According to qPCR analysis, the expression of Bcl-2 in FA groups were reduced while Bax were correspondingly increased, and the differences were statistically significant (*P* < 0.05). These alterations in various concentrations of FA groups were occurred in the concentration- dependent manner. (Fig.4)

## DISCUSSION

FA, a phenolic compound which is abundant in cereal grains, vegetables, fruits and herbs, exerts the multifarious biological activities. FA could get the antioxidant activity in human dermal fibroblasts via increasing quantities of heat shock protein-70 and heme oxygenase-1 and decreasing lipid oxidation and hydrogen peroxide-induced protein.^10^ FA exhibits anti-inflammatory activity by modulating of dendritic cells functions to restore Th1/Th2 imbalance in the asthmatic mouse model.^3^ FA may modulate the status of lipid per-oxidation and antioxidants to contribute to anti-proliferative activity during the 7,12-dimethylbenz[a] anthracene-induced skin carcinogenesis.^8^ FA shows cytotoxicity and apoptosis activity through prompting oxidative DNA breakage enhanced by endogenous copper to kill SiHa cervical cancer cells and HepG2 hepatocellular carcinoma cells without affecting normal cells.^11^ In this study, both SaOS-2 and MG63 cell viabilities were significantly decreased due to exposure to FA.

Caspases, a family of cysteine-dependent aspartate-specific proteases possessing capability of cleaving proteins at aspartic acid residues, are divided into initiator caspase and executioner caspase which are inactive without stimulant.^12^ Once initiator caspase becomes activated and directly cleaves executioner procaspase-3 into caspase-3, degradation of chromosomal DNA initiated ultimately leads to an irreversible cell death.^13^ The apoptosis triggered by FA may be associated with caspase-3 activation, since a decrease of procaspase-3 accompanied with an increase of caspase-3 are discovered in MLTC-1 tumoral cells and TM-3 Leydig cells treated FA.^14^ The apoptosis is stimulated by the significantly increased expression of caspase-3 in FA treated MIA PaCa-2 pancreatic cancer cells.^15^ It is discovered that caspase-3 was up-regulated in T24 bladder cancer cells cultured with FA.^16^ This study demonstrates the similar phenomenon in SaOS-2 and MG63 cell lines, which indicates FA promotes apoptosis through caspase-3 activation.

Bcl-2 has the capability to inhibit apoptosis through blocking the activation of the mitochondrial caspase-3 activated by various pro-apoptotic stimuli.^17^ The high expression of Bcl-2 inhibiting apoptosis may be closely associated with the degree of malignancy, lower long-term survival rate and the occurrence of osteosarcoma.^18^ The previous literature has reported that FA promotes apoptosis to display the antitumor activities through significant decreases in expression of Bcl-2 genes in LNCaP prostate cancer cells.^7^ According to the function, Bcl-2 family has another category members, like Bax, which promote the apoptosis.^19^ FA induced an increased expression of Bax accompanied with a significant decreased expression of Bcl-2 in the MIA PaCa-2 cells.^15^ It is reported that FA could increase expression of Bax with concomitant decreased Bcl-2 in the photocarcinogenesis progress of male Swiss albino mice skin.^20^ It is also discovered that Bax was prominently up-regulated while Bcl-2 was down-regulated in T24 bladder cancer cells cultured with FA.^16^ In this paper, it is demonstrated that Bcl-2 mRNA and proteins are decreased and Bax simultaneously increased in the FA treated SaOS-2 and MG63 cells compared with the control group.

## CONCLUTION

The results of this study have elucidated that FA could promote apoptosis through activated both caspase-3 and Bax and inactivated Bcl-2, and significantly descend cell viabilities in both SaOS-2 and MG63 osteosarcoma cell lines. Although the molecular mechanisms of this necessitate further studies, FA is expected to become an attractive and effective material of therapeutic strategies of osteosarcoma.
